# Microwave Diamond-Based HBAR as a Highly Sensitive Sensor for Multiple Applications: Acoustic Attenuation in the Mo Film

**DOI:** 10.3390/s23094502

**Published:** 2023-05-05

**Authors:** Boris Sorokin, Nikita Asafiev, Dmitry Yashin, Nikolay Luparev, Anton Golovanov, Konstantin Kravchuk

**Affiliations:** Federal State Budgetary Institution “Technological Institute for Superhard and Novel Carbon Materials” (FSBI TISNCM), Troitsk, 108840 Moscow, Russia; asafev.no@phystech.edu (N.A.); iashin.dv@phystech.edu (D.Y.); luparev@gmail.com (N.L.); anton.golovanov2012@gmail.com (A.G.); kskrav@gmail.com (K.K.)

**Keywords:** high overtone bulk acoustic resonator (HBAR), quality factor, microwave band, ultrasonic sensor device, microwave hypersonic attenuation, molybdenum magnetron deposition, single-crystalline diamond, aluminum–scandium nitride

## Abstract

The application of microwave diamond-based HBAR as a sensor of microwave acoustic attenuation α was considered, using the Mo film as an object of research. A multilayered piezoelectric structure, as the Al/Al_0.73_Sc_0.27_N/Mo/(100) diamond/Mo, was produced using aluminum–scandium nitride composition, and was studied in detail for a number of the Mo films with different thicknesses obtained by magnetron deposition. The operational frequency band of 3.3 … 18 GHz was used. It was found that the dependence of the resonant frequency shift vs. the *h*(Mo) thickness for all the overtones to be investigated was linear. For a given sensor, it was found that the mass sensitivity per unit area *r_m_* was equal to −26 × 10^−12^ and −8.7 × 10^−12^ g/(cm^2^∙Hz) at 6.0 GHz and 18.3 GHz, respectively. The frequency dependencies of quality factor *Q*, which changed as a result of Mo film deposition, were considered as the basic experimental data. A method for extracting the α(Mo) values was proposed. The *Q*-factor under the complete deposition of Mo film was 936 nm, and dropped moderately to ~25%. Such values were enough for an aim of the given experiment. The α(*f*) in molybdenum was obtained, and demonstrated a dependence that was close to quadratic, corresponding to the Akhiezer attenuation law.

## 1. Introduction

There are a number of approaches to developing sensory devices intended for use in many applications, e.g., to measure gas concentration, humidity, and thin film properties; to investigate viruses and other microbiologic objects, to develop accelerometers, etc. The miniature acoustoelectronics sensors have a specific niche due to their high sensitivity, compatibility with the planar microelectronic technology, low cost per unit, and resistance in harsh environment conditions.

Most of the known acoustic sensory methods are based on the changes in the resonant frequency and quality factor *Q* of an oscillating plate, a silicon cantilever, and widely used surface acoustic wave (SAW) devices or resonators on bulk acoustic waves (BAW). A prospective method for the design of ultra-sensitive devices is related to the technology used in nano- or microelectromechanical systems (NEMS, and MEMS). For example, the smallpox virus mass, 12 fg, was found using both a microscale silicon cantilever and atomic force microscopy [1]. A demonstration of Au films’ mass deposition of the resolution of some attograms measured by a NEMS sensory device is presented in the paper [2]. The authors of the paper referenced in [3] developed and investigated a novel resonant MEMS sensor for the detection of dielectric particles in liquids. The responsivity and limit of detection of the measured mass were in the ranges of 13 Hz/ng and 3.5 ng, respectively. The fabrication and electrical properties of the single-crystal silicon MEMS resonator with a side length of 1 mm and a thickness of ~40 μm (1 mm × 1 mm × 40 μm), operating at Lamé and extensional modes, is presented by authors of [4]. This device was applied as a sub-ng mass resolution sensor of airborne particles in a flow. However, such unique sensors should operate in precise environmental conditions. As a rule, the MEMS (NEMS) operating frequency is limited to a band of several MHzs, and cannot be further increased. A MEMS (NEMS) sensory element is not designed for multiple uses. Thus, the search for analogs which are simpler and more convenient in a practical sense remains open. It should be highlighted that a fine-quality sensor has to be designed according to a principle combining a high operating frequency with high *Q*, which is not often realized.

The most widely employed sensor is the quartz crystal microbalance (QCM), which was first developed by Sauerbrey [5]. As a basic component of QCM, a conventionally high *Q* quartz piezoelectric resonator was used. Longitudinal (L) or shear (S) bulk acoustic waves were applied as operational modes. Sauerbrey proposed that the mass per unit area loaded on a quartz crystal surface was proportional to the resonance frequency shift. Such a dependence is rightful if a rigid film is uniformly placed on the surface and its thickness is less than that of the quartz crystal plate. Studying the metal film deposition, it was shown that the reduced mass sensitivity of a QCM changes from 10^−8^–10^−10^ g/(cm^2^∙Hz) [6]. It was also shown that when loading a large mass, Δ*m*, the dependence between frequency shift and Δ*m* becomes more complicated. To explain this, the ratio of the acoustical impedances of quartz and deposited film should be taken into account. The authors of the review in [7] observed many QCM practical applications, such as studying metal film deposition, etching electroplated alloys, the oxidation processes of metal films, and the investigation of the physical properties of solid–liquid interfacial layers in situ. Typically, the maximal operating frequency of QCM, designed as an inverse mesa structure, is limited by several hundreds of MHz. Further plate thinning, up to several microns, leads to a loss of mechanical toughness and a decrease *Q*-factor.

SAW sensors based on resonators and delay lines are also widely applied. Piezoelectric substrates produced of ST-cuts of quartz, LiNbO_3_, LiTaO_3_, langasite, langatate, etc., allow for the typical operational frequency band of several hundred MHz to be obtained. As usual, the mass sensitivity of SAW sensors has a value of about 2.5 × 10^−9^ g/(cm^2^∙Hz) [8]. It should be noted that the open area of a substrate between two interdigital transducers (IDT) is highly sensitive to various actions, such as pressure, film deposition, humidity, gas absorption by sensitive films, and other many bias fields. The maximal operating frequency of such devices is limited by the units of GHz due to air loading, which leads to the *Q*-factor decreasing by up to several hundred units.

The gravimetric sensor used as a component the film bulk acoustic resonator (FBAR) had a typical mass sensitivity about 3.6 × 10^−6^ g/(cm^2^∙Hz) [9]. For example, at the Ti film deposition, this sensor showed a thickness sensitivity about 1.6 nm at the operating frequency of 2442 MHz. Using MEMS technology, the authors of [10] developed an FBAR-based mass sensor, built on a silicon nitride diaphragm. The sensor could be operated in both vapor and liquid media, and had a mass sensitivity of 726 cm^2^/g. In the authors’ opinion, this value was about 50 times greater than that of a typical QCM sensor. However, the *Q*-factor in the air was ~200–300 units at 1 GHz, which then dropped to 40 at 2 GHz. As a result, the sensor’s sensitivity decreased drastically at the microwave frequency band. A more convenient and efficient device is the so-called solidly mounted resonator (SMR), in which a thin-film acoustic resonator is deposited on a Bragg reflective grating placed on the Si substrate. Authors of paper [11] used, as a sensor of organic films, the SMR included the build-in FBAR with the Pt/AlN/Pt structure. A fundamental resonant frequency of ~6 GHz was obtained, owing to the small AlN thickness of 180 nm. As an investigation object, the 11-mercaptoundecanoic acid was selected. The shift in resonant frequency under the acid mass deposition on the top Pt electrode was 6 MHz, and the *Q*-factor dropped from 183 to 177. The authors [12] used an FBAR-based sensor with a shear BAW mode for the purpose of studying the properties of liquids. From the reference value of *Q* ~ 300 in air at a resonant frequency of 1.25 GHz, the *Q*-factor decreased to 150 units in water. It should be noted that both FBAR-based sensors and those developed using MEMS or NEMS technology are unlikely to be reused due to the fragility of their structure.

Mansfeld et al. proposed the high overtone bulk acoustic resonator (HBAR) with Me/ZnO/Me/YAG structure as a sensor to study metal films [13]. Acoustic properties such as the phase velocity and microwave acoustic attenuation in the W, Ti, Mo, and Al thin films were studied. The sensory properties of two HBAR structures, “AlN/SiO_2_” and “YX*l*/163°LiNbO_3_/YX*l*/32° quartz”, were investigated both by experimental and model methods [14]. L- or S-waves are used as the operational modes, respectively. The authors proposed improving the HBAR gravimetric (mass) sensitivity. The BAW reflection and penetration into the films were also studied.

Hence, owing to the HBAR’s high *Q*-factor and high operational frequency in a microwave band, its sensitivity can be increased compared with other kinds of acousto-electronic sensors. Such a useful combination significantly exceeds that observed with the SAW- or FBAR-based sensors. However, in our opinion, the HBAR’s sensory properties, including the low number of objects investigated, are insufficiently studied to date. The increased sensitivity can be realized only if the HBAR substrate’s material possesses low acoustic attenuation at a microwave band. Recently, we [15] achieved excitation of the diamond-based HBAR up to 40 GHz, with *Q* ~10,000 units, at room temperature. The diamond’s excellent acoustic properties were investigated in the super high (SHF) and extremely high frequency (EHF) bands. As was shown in the paper [16], the Landau–Rumer mechanism of microwave acoustic attenuation α in the diamond predominated at frequency bands of more than 1.5–2 GHz. In this case, the frequency dependence was α(*f*)~*f*, while the *Q*-factor remained at a constant value. As a result, an HBAR excitation up to the EHF band became possible. It is important to highlight that the aluminum–scandium nitride Al_1-*x*_Sc*_x_*N (ASN) piezoelectric film was provided with L-type acoustic wave excitation in a frequency band up to 40 GHz [15]. Earlier, diamond-based HBAR sensors using the “Me1/(Al,Sc)N/Me2/(100) diamond/Me3” and “Me1/AlN/Me2/(100) diamond/Me3” structures were applied to study the thin and ultrathin metal films such as Mo, Al, Sc, and Pt [17,18,19,20]. The thickness sensitivity under the Pt deposition was estimated as ±0.5 nm.

The HBAR sensors have a wide variety of applications; yet, the number of articles dedicated to them was unremarkable for the last ten years. The studies in which HBAR-based devices were applied as gravimetric sensors for solid films continued the works of Mansfeld [13]. In these works, the authors tried to increase the sensitivity by raising operational frequencies [20] up to 20 GHz, implementing different substrates and piezoelectric layers [14]. During these studies, a model for calculating frequency shifts with more precision than Sauerbrey’s model was suggested. On the other hand, the number of experiments with the liquid and semi-liquid substances was high. A viscosity sensor utilizing shear waves, with good agreement between the theoretical model and the experiment, was developed by Yamakawa [21]. Different approaches to the detection of liquid in micro-trenches were studied by Yanez [22,23]. The authors of the work [23] also showed that HBAR could not only be successfully utilized as a resonator, but also could operate in an echo-pulse mode. These authors also presented the possibility of creating a reliable fingerprint scanner based on the array of HBARs with 366 dpi resolution. However, some specified approaches to utilizing HBAR exist, such as a preventing electronic scavenging wireless sensors [24], or hybrid sensors utilizing HBAR as a switch for NV-centers in diamond [25]. As one can see, the potential to implement HBAR as a sensor is promising, but this field is still understudied.

In our opinion, the level of investigation on microwave acoustic attenuation in thin films, which are the important components of acousto-electronic devices, has been unsatisfactory to date. Thus, this work makes the following contributions to this field: (i) the novel diamond-based HBAR is developed to obtain an operational frequency of the sensor close to 20 GHz; (ii) the experimental *Q*-factor and frequency shift dependencies vs. the Mo film thickness obtained in the enhanced frequency band up to 18.3 GHz are introduced and discussed; (iii) a method of extracting microwave acoustic attenuation of the Mo metal film from the experimental data on the total *Q*-factor in a wide frequency band is proposed.

The main objective of this work concerns the detailed study of microwave acoustic attenuation in the Mo metal film by the diamond-based HBAR within an enhanced frequency band.

## 2. Materials and Methods

For a declared purpose, the “Al/Al_0.73_Sc_0.27_N/Mo/(100) diamond/Me” diamond-based multilayer piezoelectric structure (MPS), as a sensory device, was developed by a known technology using magnetron sputtering [20]. First, the conducting Mo layer was deposited onto a cleaned diamond surface. To obtain an excitation of L-type acoustic waves, the piezoelectric film, as Al_0.73_Sc_0.27_N (ASN) with (0002) orientation of crystallites, was synthesized using the AJA Orion 8 rf magnetron sputtering equipment. The high-purity Al and Sc metals were sputtered simultaneously in a flow of Ar/N_2_ gas mixture. The plasma-forming gas Ar was delivered directly into the magnetron, and the reactive gas N_2_ was delivered to the area around the substrate. To obtain the required mixture, the Al and Sc magnetron powers were equal to 300 and 117 W, respectively. The Sc content was proven using the XRD method and data on the dependence between unit cell parameters and the Sc/Al ratio published in the paper [26]. The Al_0.73_Sc_0.27_N film grew at a rate of about 100 nm/hour. The lift-off photolithography technique was used with the Heidelberg μPG-101 direct laser lithography apparatus to create the contacts and piezoelectric layer of a specific topology.

As a substrate material, a synthetic IIa-type single-crystalline diamond, grown at the Technological Institute for Superhard and Novel Carbon Materials using the temperature gradient method under high-pressure and high-temperature conditions, was used [27]. The IIa type diamond differs due to its low content of nitrogen point defects and superior dielectric and elastic properties. The double-sided substrate faces were prepared with deviation better than 5′ from the [100] diamond crystalline direction, and were polished to obtain roughness *R_a_* better than 10 nm/100 microns. The roughness was controlled using the AFM method. The main parameters of the developed sensor are shown in Table 1. Across the working MPS area, a number of diamond-based HBARs with different active zones (apertures) could be located. Effective excitation of the acoustic wave in HBAR is concerned with the area of the resonator’s aperture. The higher the operational frequency used, the lower the aperture should be. In our experiment, we used the diamond-based HBAR with an aperture area of ~10,000 sq. microns, which provided a good level of acoustic signal from 1 to 20 GHz. As an object of research, the Mo films of various thicknesses were chosen. This choice could be explained by a close accordance in the acoustic impedances of the diamond substrate and molybdenum, as *Z*_diam_ = 61.7 and *Z*_Mo_ = 63 MRayl. In this case, the acoustic wave propagation through the diamond/Mo interface should be free in practice because the reflection coefficient is equal to *R* ≈ 0.01. One can say that the diamond substrate has its own extension of an acoustic path. It can be assumed that after the Mo deposition, the *Q*-factor should be changed due to acoustic attenuation in the deposited material.

**Figure 1:**
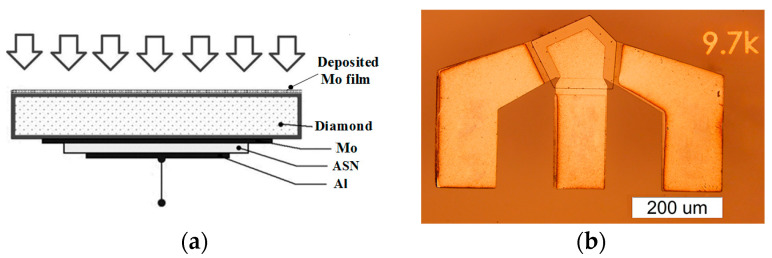
(**a**) The scheme of Mo film deposition on the free side of the diamond-based HBAR sensor. (**b**) The bottom view of the “Al/Al_0.73_Sc_0.27_N/Mo/(100) diamond” sensor to be used.

An important feature of the chosen sensory scheme (Figure 1a) is that the studied Mo film was deposited onto the free surface of the diamond-based HBAR, and did not have a direct effect on the Al/ASN/Mo thin-film piezoelectric transducer (TFPT), which excites the operating L-mode in the diamond. Thus, the surface of the diamond with the TFPT was completely protected from any damage, and the sensor could be used for an unlimited number of experiments. In the interval between studies, the working surface of the diamond could be easily cleaned without damage due to its hardness. The photograph shown in Figure 1b represents a bottom view of the “Al/Al_0.73_Sc_0.27_N/Mo/(100) diamond” sensor to be used. Notice that the lateral size of a TFPT aperture is close to ~100 microns. Since the hypersonic beam of a wave is strictly limited by the aperture, this miniature sensor can be used for local measurements of deposited objects. As one can see, the electrode arrangement responded to the G-S-G connection scheme. The pentagonal form of the bottom Al-electrode placed on the ASN surface helped to suppress parasitic peaks, known as inharmonics, and to improve the useful signal peak in a certain frequency band. A step-by-step process was used to deposit the Mo films of different thicknesses on the free side of the diamond substrate by the AJA Orion 8 magnetron sputtering equipment. The speed of Mo deposition was equal to ~9.3 nm/min, and the Ar pressure was equal to 5 mTorr. The number of Mo layers was equal to 10, and the mean thickness of each was equal to ~93 nm. For precise thickness measurement, the atomic force microscope (AFM) method was applied for a number of Mo/Si test samples using Ntegra-Prima equipment. The thickness uncertainty obtained by the AFM was changed from 10 to 3 nm.

After every step of the Mo deposition onto the free side of the diamond substrate, the sensor was investigated in the one-port mode using the E5071C Agilent network analyzer (300 kHz–20 GHz) and the M150 probe station, providing the G-S-G connection of a sensor. All of the SHF measuring procedures were carried out at a stabilized room temperature of 25 °C. First, the frequency response measurement consisted of obtaining *S*_11_(*f*) dependence, where *S*_11_ is the complex reflection coefficient. Then, using *S*_11_(*f*) data, the real part, Re*Z*_11e_(*f*), taken as a signal, was calculated. Here, *Z*_11e_(*f*) = (Re*Z*_11_ − Re*Z*_11*b*_) + *i*(Im*Z*_11_ − Im*Z*_11*b*_) is the so-called extracted impedance, *Z*_11_ is the total electric impedance, and the quantities Re*Z*_11*b*_ and Im*Z*_11*b*_ are outside a given operational frequency *f_m_* of the *m*-th acoustic overtone. The shift of resonant frequency Δ*f_m_* and quality factor *Q_m_*(*f*) for a chosen overtone were measured after every new stage of Mo deposition. A conventional method determining the *Q*-factor at the −3 dB level relative to the maximum of the Re*Z*_11e_(*f*) impedance peak was used. See the paper cited in [16] for more information on microwave measurement details.

**Figure 2:**
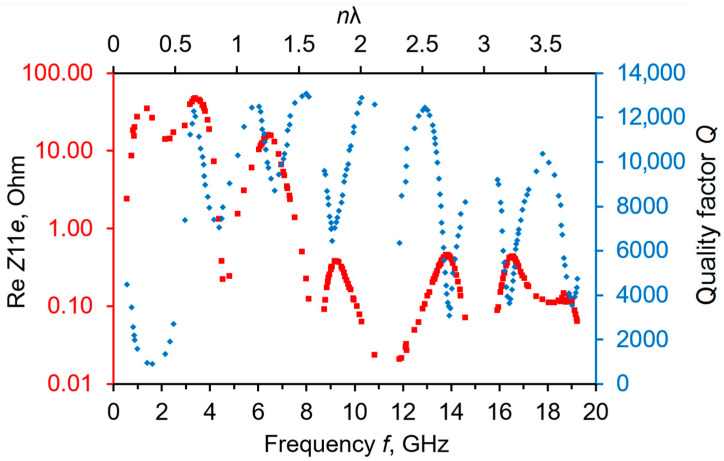
The reference state of a frequency response of the Al/Al_0.73_Sc_0.27_N/Mo/(100) diamond sensor where the dependencies of Re*Z*_11e_(*f*) (in red) and *Q*(*f*) (in blue) are shown. The top horizontal axis corresponds to values of an effective acoustic wave length in the TFPT.

Figure 2 represents the initial frequency response of a given sensor, including Re*Z*_11e_(*f*) and *Q*(*f*) dependencies within the 1–19 GHz bands. Minimums in the *Q*(*f*) curves are observed at the points when *n*λ/4 ≈ *h*_ASN_, and *n =* 1, 3, … On the contrary, the *Q* maximums arise when *n*λ/2 ≈ *h*_ASN_; λ is the acoustic wave length in the ASN film; and *n =* 1, 2, 3, etc. All of these peculiarities connect with the proper characteristic frequencies of the Al/ASN/Mo TFPT in the process of its interaction with the diamond substrate. As a rule, the higher the Re*Z*_11e_ values, the lower the *Q*-factors at the same operational frequency points, and vice versa. The overtones with the highest quality factors were selected as operational modes. Note that the *Q*(*f*) maximums at 3, 6, 8, 10, and 13 GHz have approximately the same values, i.e., ~13,000 units. This fact corresponds with the Landau–Rumer acoustic attenuation mechanism observed in the diamond for the L-BAW mode above 1.5–2 GHz [16].

The operational checkpoint frequencies were chosen in the band of 3 up to 18 GHz. When the measurement of microwave acoustic parameters for the *p*-th Mo layer was over, the sensor returned them to the magnetron chamber, the #*p +* 1 layer was sputtered, and then the measurement process was repeated.

## 3. Results

**Figure 3:**
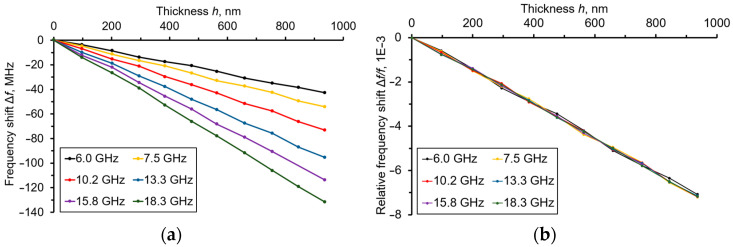
(**a**) The Δ*f* vs. the *h*(Mo) dependencies and (**b**) Δ*f*/*f* vs. the *h*(Mo) dependencies, measured at resonant frequencies from 6.0 to 18.3 GHz of the acoustic overtones taken as operational modes by the “Al/Al_0.73_Sc_0.27_N/Mo/(100) diamond/Mo” sensor.

After sequential deposition of the Mo film, the microwave measurements of a number of parameters, such as the Re*Z*_11*e*_, Δ*f* absolute frequency shifts and f/f relative ones, and Qn was fulfilled. All of the experiments were performed at room temperature, stabilized at about 25 °C. The AFM measurement of the *h*(Mo) film thickness was performed using Mo/Si test samples, which are produced under the same conditions as the “Al/Al_0.73_Sc_0.27_N/Mo/(100) diamond/Mo” sensor. Figure 3 represents the Δ*f* and Δ*f*/*f* dependences vs. *h*(Mo) measured for many of the resonant frequencies of acoustic overtones. One can see that up to the maximal thickness of *h*(Mo) = 936 nm, there was a proportional drop in the resonant frequencies vs. the *h*(Mo) increment. In this case, the maximal frequency shift for the 18.3 GHz overtone was −140 MHz. The slopes of all the curves were the same for all overtones measured from 6 to 18.3 GHz, and were equal to −7.8 × 10^−6^ nm^−1^ (Figure 3b). This confirms the suggestion that the deposited Mo film serves as a prolongation of an acoustic path on the diamond substrate.

Because of Δ*f*/*f*~Δ*h*, the relations and definitions of the sensor sensitivity parameters proposed for the QCM and FBAR-based sensors in the papers cited in [28,29,30] could be applied in the case of Mo film deposition on a diamond-based HBAR sensor as well. Thus, for an additional analyzed layer with a density of ρ*_m_* and a film thickness of *h_m_* deposited on the sensor, the relative shift in the resonant frequency was calculated using the equation:

(1) $$\frac{\Delta f}{f}\approx -\frac{\rho_{m}h_{m}}{\rho_{0}h_{0}}=-\frac{\Delta m}{m_{0}}$$

where Δ*m* is the additional mass per unit area; and ρ_0_, *h*_0_, and *m*_0_ are the density, thickness, and mass (per unit area) of the sensor, respectively. The minus sign determines the decrease in frequency along with the increase in mass load. Equation (1) is valid if a Δ*m* is less than 2% of a sensor’s initial mass *m*_0_. In the case of a distributed mass sensor, the mass sensitivity (in cm^2^/g units) is defined as:

(2) $$S_{m}=\underset{\Delta m\to 0}{\lim}\left(\frac{\Delta f}{f}\right)\left(\frac{1}{\Delta m}\right).$$

Using the Relations (1) and (2), one can obtain the formula:

(3) $$S_{m}=-\frac{1}{\rho_{0}h_{0}}.$$

For the HBAR used in this work, we obtained *S_m_* = −6.3 cm^2^/g. It should be noted that the *S_m_* value depends on the substrate material properties as well as the *h*_0_ sensor thickness. Thus, if we take a lower *h*_0_ value, it is easy to obtain a higher *S_m_* parameter. For example, when *h*_0_ = 0.01 cm, the *S_m_* = −31.6 cm^2^/g. The second important parameter is the minimal frequency response of the sensor, defined as:

(4) $$R_{f}=\frac{\Delta f_{\min}}{f}$$

where $\Delta f_{\min}$ is the minimum detectable frequency shift. Estimations of the parameter *R_f_* for a given sensor with a deposited Mo film 0.5 nm in thickness, close to the physical limit of detection, resulted in a value of *R_f_* = 3.2 × 10^−6^. In this case, the appropriate Δ*f_min_* was equal to −19.2 and −58.6 kHz at 6.0 and 18.3 GHz, respectively. The real limit of resolution for the measuring system utilized herein, including HBAR&E5071C, was equal to ~5 kHz. Therefore, such a resolution allows for easy use of a system to study the ultra-thin film deposition of metals with lower densities. The minimum detectable change in mass per unit area is calculated from relation (2), and has the form:

(5) $$\Delta m_{\min}=\frac{R_{f}}{S_{m}}.$$

Mass sensitivity per unit area (in the g/(cm^2^·Hz) units) can be calculated using the following formula:

(6) $$r_{m}=\frac{1}{fS_{m}}.$$

For a given sensor, it was calculated that the mass sensitivity per unit area *r_m_* was equal to −26 × 10^−12^ and −8.7 × 10^−12^ g/(cm^2^∙Hz) at 6.0 GHz and 18.3 GHz, respectively.

**Figure 4:**
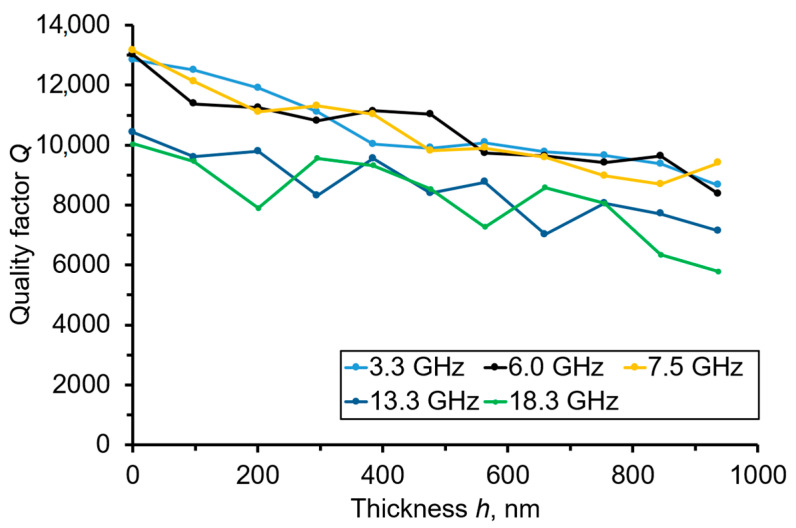
The *Q_n_* vs. the *h*(Mo) dependencies, measured at resonant frequencies from 3.3 to 18.3 GHz, of overtones observed in the “Al/Al_0.73_Sc_0.27_N/Mo/(100) diamond/Mo” sensor.

Figure 4 represents the *Q_n_* dependencies vs. *h*(Mo) obtained as a result of the Mo film deposition and measured at the microwave operational frequencies from 3.3 to 18.3 GHz. All of the dependencies were close to the linear ones. It should be noted that the *Q*-factor, under the complete deposition of the Mo film, i.e., 936 nm, dropped moderately to ~25%. Such values were enough for the aim of the given experiment.

The general equation for the amplitude of an attenuating acoustical wave passing along the *x* direction is:

(7) $$A_{x}=A_{0x}\exp\left(-\alpha h\right)\exp i\left(\omega t-kx\right)$$

where $A_{x}\;and\;A_{0x}$ are the current and initial amplitudes, respectively; α is the attenuation (in Np/cm); *h* is the thickness of the material; ω is the angular frequency; *t* is the travel time; and *k* is the wave number. The exp[*i*(ω*t* – *kx*)] multiplier was further omitted. Then, the amplitude of acoustical wave passed through all of the HBAR’s layers was rewritten as:

(8) $$A=A_{0}exp\left(-\alpha_{Al}h_{Al}\right)exp\left(-\alpha_{ASN}h_{ASN}\right)exp\left(-\alpha_{Mo}h_{Mo}\right)exp\left(-\alpha_{diam}h_{diam}\right)$$

where $\alpha_{Al}\;and\;h_{Al},\alpha_{ASN}\;and\;h_{ASN}$, $\alpha_{Mo}\;and\;h_{Mo}$, and $\alpha_{diam}\;and\;h_{diam}$ are the attenuations and thicknesses of each layer in the HBAR as top Al electrode, ASN piezoelectric film, bottom Mo electrode, and diamond substrate, respectively.

The whole thickness of the HBAR is equal to:

(9) $$h_{tot}^{0}=h_{diam}+h_{Mo}+h_{ASN}+h_{Al}.$$

The total attenuation along the whole resonator, taken in Np, could be stated as:

(10) $$\alpha_{tot}^{0}h_{tot}^{0}=\alpha_{diam}h_{diam}+\alpha_{Mo}h_{Mo}+\alpha_{ASN}h_{ASN}+\alpha_{Al}h_{Al}.$$

Thus, the amplitude of the acoustic wave passing through the HBAR has the form:

(11) $$A=A_{0}\exp\left(-\alpha_{tot}^{0}h_{tot}^{0}\right).$$

After the deposition on the diamond-free side of a Mo layer with the thickness $h\left(Mo\right),$ the new total attenuation taken in Np in the HBAR + Mo system had the following form:

(12) $$\alpha_{tot}h_{tot}=\alpha_{Mo}h\left(Mo\right)+\alpha_{tot}^{0}h_{tot}^{0}.$$

Since the acoustical impedances of Mo and the diamond were almost equal [31], the reflection coefficient at the diamond/Mo interface could be estimated as ~0.01, and the effect of wave reflection had not been taken into consideration. As a result, the amplitude of the wave was be changed to:

(13) $$A\left(Mo\right)=A_{0}\exp\left[-\alpha_{Mo}h\left(Mo\right)\right]\exp\left(-\alpha_{tot}^{0}h_{tot}^{0}\right).$$

The acoustical attenuation α vs. the *Q*-factor was defined in agreement with the monograph [32]:

(14a) $$\alpha =\frac{\pi f}{QV}\;\left[Np/cm\right],$$

(14b) $$\alpha =\frac{\pi f}{Q}\;\left[Np/s\right]$$

where *f* (in Hz) is the resonant frequency and *V* (in cm/s) is the phase velocity of a material. Let us suppose that the effective phase velocity of the HBAR and HBAR + Mo systems can be calculated as:

(15) $$V_{tot}^{0}=\frac{1}{h_{tot}^{0}}\left({{h_{Al}V_{Al}+h}_{ASN}V_{ASN}+h}_{Mo}V_{Mo}+h_{diam}V_{diam}\right),$$

(16) $$V_{tot}=\frac{1}{h_{tot}}\left\{{{h_{Al}V_{Al}+h}_{ASN}V_{ASN}+[h}_{Mo}+{h(Mo)]V}_{Mo}+h_{diam}V_{diam}\right\}.$$

Using relations (15), (16), (14), and (12), one can obtain the formula:

(17) $$\alpha_{Mo}\left[Np/cm\right]=\alpha_{tot}\left(1+\frac{h_{tot}^{0}}{h(Mo)}\right)-\alpha_{tot}^{0}\frac{h_{tot}^{0}}{h(Mo)}.$$

Often, it is convenient to define the α values in dB/cm or dB/μs units. In order to obtain the attenuation α in the above units, the following relations should be used:

(18a) $$\alpha \left[dB/cm\right]=20\mathrm{lg}\left(e\right)\cdot \alpha \left[Np/cm\right]\approx 8.686\alpha \left[Np/cm\right],$$

(18b) $$\alpha \left[\mathrm{dB}/\mu s\right]=10^{-6}\alpha \left[dB/cm\right]\cdot V\left[cm/s\right].$$

**Figure 5:**
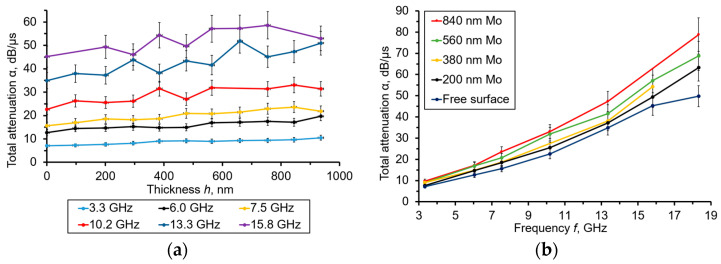
(**a**) The calculated α (in dB/μs) vs. the *h*(Mo) thickness dependencies obtained at resonant frequencies from 3.3 to 15.8 GHz. (**b**) The frequency dependencies of calculated (in dB/μs), obtained under the several thicknesses of Mo film.

Figure 5 represents the dependences of the calculated acoustic attenuation α (in dB/μs) vs. the *h*(Mo) thickness obtained at different resonant frequencies, as well as the frequency dependences of the calculated α (in dB/μs) obtained under the several thicknesses of Mo film.

**Figure 6:**
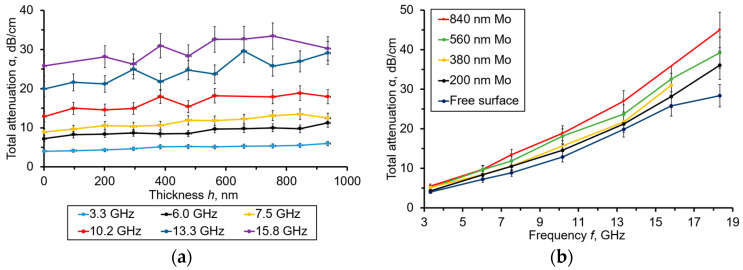
(**a**) The calculated α (in dB/cm) vs. the *h*(Mo) dependencies obtained at resonant frequencies from 3.3 to 15.8 GHz. (**b**) The frequency dependencies of calculated (in dB/cm), obtained under the several thicknesses of Mo film.

By analyzing the curves shown in Figure 5a, one can observe a slight increment of α vs. that of *h*(Mo). The attenuation also grew at higher operational frequencies. The noticeable dispersion of the result observed at the highest frequencies could be explained by the signal/noise decrease. The α(*f*) curves in Figure 5b demonstrate dependencies close to those of the α~*f*^2^ when the Mo film was deposited, while, in the case of a free diamond surface, this dependence is close to a linear one. The first kind of a dependence is associated with the Akhiezer attenuation law. The second one satisfies the Landau–Rumer law, which has been established for single-crystalline diamonds at microwave frequencies higher than 1–1.5 GHz [16]. In Figure 6, the appropriate data regarding α are presented in dB/cm units.

**Figure 7:**
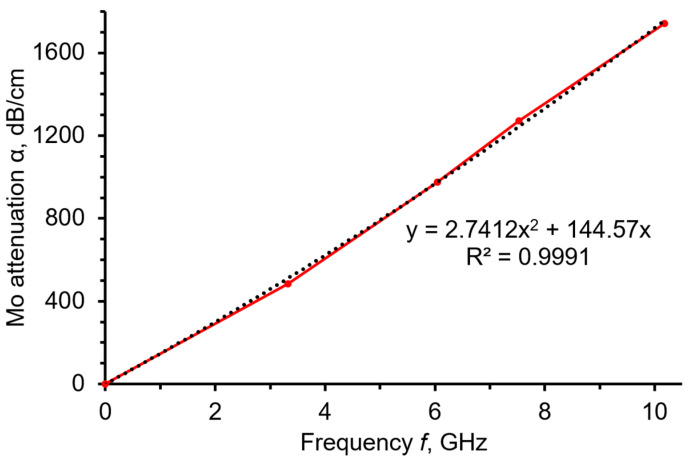
The calculated frequency dependence of microwave attenuation α (in dB/cm) in the molybdenum film deposited on the “Al/Al_0.73_Sc_0.27_N/Mo/(100) diamond/Mo” sensor.

Figure 7 represents the α(*f*) dependence extracted for molybdenum using the relations (17) and (18a). As one can see, the α(*f*) is close to a quadratic dependence, corresponding to the Akhiezer attenuation law.

## 4. Discussion and Conclusions

The authors demonstrated the possibility of utilizing the diamond-based HBAR as a microwave acoustic attenuation sensor in molybdenum within the wide frequency band from 3 to 18 GHz at room temperature. The technique of the step-by-step magnetron deposition was used to obtain a final Mo thickness of 936 nm. The dependencies of the resonant frequency shift vs. the *h*(Mo) thicknesses for all the overtones were investigated and were linear. This confirms the suggestion that the Mo film serves as a prolongation of the acoustic path on a diamond substrate, because the acoustical impedances of a Mo and diamond are almost equal to one another.

For a given sensor, the mass sensitivity per unit area *r_m_* was equal to −26 × 10^−12^ and −8.7 × 10^−12^ g/(cm^2^∙Hz) at 6.0 GHz and 18.3 GHz, respectively. This value was the better of the known SAW- and QCM-based sensors. A high-resolution, unique FBAR-based mass sensor, developed by the authors of [10] using MEMS technology, is concerned with ultra-thin piezoelectric film. However, the QCMs’ and FBARs’ operational frequencies are physically restrained due to the limited thickness of the layers and, thus, limited *r_m_* values. On the other hand, the sensitivity of an HBAR-based mass sensor could be easily improved by several times by decreasing the thickness of the sensor substrate. Additionally, the application of the highest operational frequencies of HBAR provides a possibility to decrease the relative shift in the resonant frequency. The last is one of the main parameters of an HBAR-based sensor. In this study, diamond-based HBAR was excited on an operational frequency of 40 GHz, and there is space to improve this.

In a given experiment, the instrumental error in the frequency measurement is about 5 kHz. Since measurements were run within the same temperature of 25 ± 1 °C, the random error was about 20 kHz. In this work, the smallest measured frequency shift value was equal to ~2.5 MHz. Thus, the relative error ε*_f_* was less than 1%. However, the quality factor *Q* measurements are less precise, and ε*_Q_* varies from 5 to 10%. The absolute error of the Mo thickness is caused by AFM measurements, and equals ±9 nm in a given case. The uncertainty of the indirect measurements was calculated by the appropriate formulas.

It was found that the α(*f*) in molybdenum film is close to a quadratic dependence, corresponding to the Akhiezer attenuation law. Mansfeld et al. [13] measured the attenuation of metal films, including Mo, by the method of HBAR spectroscopy. However, a direct comparison with our data would be incorrect, because we used significantly higher operational frequencies. Authors hope that the method of microwave attenuation investigation using an HBAR sensor could be applied to study the microwave attenuation properties of various thin films. The obtained data will be important in the design of prospective microwave acoustic devices and sensors. Future research in this field will be concerned with the improvement of an HBAR sensor method and technique in the directions of a signal/noise increasing and an extension of operational frequency band.

We also wish to emphasize that our sensors are much more durable and resistant than any SAW-, FBAR-, MEMS-, and QCM-based sensors. The diamond-based HBAR sensor possesses some advantages over other kinds of acousto-electronic sensors due to the application of operational frequencies in the microwave band up to 20 GHz. As a result, this method has enhanced mass sensitivity; high chemical and biological inertness of the working diamond surface; resistance to temperature load; abrasive wear resistance; and unlimited, multiple uses. Owing to the miniature size of the considered sensor, it can be used for local measurements of deposited objects, possibly in the manufacturing process of nanoelectronic devices, the investigation of micro-biological objects and chemical reactions in vivo, etc. Other kinds of sensors cannot tolerate high pressure or aggressive media such as the SF_6_ plasma, which is highly used in Si etching. The attenuation measurements are highly useful for fundamental research on thin films. Now, measurements using a diamond-based HBAR sensor can be fulfilled in wide frequency ranges, from hundreds of MHz to several tens of GHz, with the same sensor. The latter allows for the establishment of a possible dispersion of material properties.

## Figures and Tables

**Table 1:** Parameters of the sensor investigated.

Top electrode material/thickness (in nm)	Al/110
Bottom electrode material/thickness (in nm)	Mo/140
ASN piezoelectric layer thickness (in nm)	1550
Diamond substrate thickness (in microns)	478
Area of the HBAR aperture (in sq. microns)	~10,000

## Data Availability

The raw data that support the findings of this study are available from the corresponding author and may be shared with the scientific communities.

## References

[1] Johnson L., Gupta A.K., Ghafoor A., Akin D., Bashir R. (2006). Characterization of vaccinia virus particles using microscale silicon cantilever resonators and atomic force microscopy. Sens. Actuators B Chem..

[2] Ekinci K.L., Huang X.M.H., Roukes M.L. (2004). Ultrasensitive nanoelectromechanical mass detection. Appl. Phys. Lett..

[3] Patocka F., Schneidhofer C., Dörr N., Schneider M., Schmid U. (2020). Novel resonant MEMS sensor for the detection of particles with dielectric properties in aged lubricating oils. Sens. Actuators B Phys..

[4] Soysal U., Marty F., Géhin E., Motzkus C., Algré E. (2020). Fabrication, electrical characterization and sub-ng mass resolution of sub-μm air-gap bulk mode MEMS mass sensors for the detection of airborne particles. Microelectron. Eng..

[5] Sauerbrey G. (1959). Verwendung von Schwingquarzen zur Wägung dünner Schichten und zur Mikrowägung. Z. Physik..

[6] Lu C.S., Lewis O. (1972). Investigation of film-thickness determination by oscillating quartz resonators with large mass load. J. Appl. Phys..

[7] Qiao X., Zhang X., Tian Y., Meng Y. (2016). Progresses on the theory and application of quartz crystal microbalance. Appl. Phys. Rev..

[8] Wen W., Shitang H., Shunzhou L., Minghua L., Yong P. (2007). Enhanced sensitivity of SAW gas sensor coated molecularly imprinted polymer incorporating high frequency stability oscillator. Sens. Actuators B Chem..

[9] Lin R.-C., Chen Y.-C., Chang W.-T., Cheng C.-C., Kao K.-S. (2008). Highly sensitive mass sensor using film bulk acoustic resonator. Sens. Actuators A Phys..

[10] Zhang H., Kim E.S. (2005). Micromachined acoustic resonant mass sensor. J. Microelectromech. Syst..

[11] Rey-Mermet S., Lanz R., Muralt P. (2006). Bulk acoustic wave resonator operating at 8 GHz for gravimetric sensing of organic films. Sens. Actuators B.

[12] Wingqvist G., Bjurström J., Liljeholm L., Yantchev V., Katardjiev I. (2007). Shear mode AlN thin film electro-acoustic resonant sensor operation in viscous media. Sens. Actuators B.

[13] Mansfeld G.D., Alekseev S.G., Kotelyansky I.M. Acoustic HBAR spectroscopy of metal (W, Ti, Mo, Al) thin films. Proceedings of the 2001 IEEE Ultrasonics Symposium. Proceedings. An International Symposium (Cat. No.01CH37263).

[14] Rabus D., Friedt J.M., Ballandras S., Baron T., Lebrasseur É., Carry É. (2015). High-overtone bulk-acoustic resonator gravimetric sensitivity: Towards wideband acoustic spectroscopy. J. Appl. Phys..

[15] Sorokin B.P., Asafiev N.O., Kvashnin G.M., Scherbakov D.A., Terentiev S.A., Blank V.D. (2021). Toward 40 GHz excitation of diamond-based HBAR. Appl. Phys. Lett..

[16] Sorokin B.P., Telichko A.V., Kvashnin G.M., Bormashov V.S., Blank V.D. (2015). Study of microwave acoustic attenuation in a multifrequency bulk acoustic wave resonator based on a synthetic diamond single crystal. Acoust. Phys..

[17] Sorokin B.P., Kvashnin G.M., Novoselov A.S., Burkov S.I., Shipilov A.B., Luparev N.V., Aksenenkov V.V., Blank V.D., Vassiliadis S.G., Matsouka D. (2018). Application of thin piezoelectric films in diamond-based acoustoelectronic devices. Piezoelectricity—Organic and Inorganic Materials and Applications.

[18] Sorokin B.P., Kvashnin G.M., Luparev N.V., Asafiev N.O., Scherbakov D.A. (2020). Studying microwave acoustic sensors based on synthetic diamond substrates. Izvestiya Vysshikh Uchebnykh Zavedenii Seriya Khimiya i Khimicheskaya Tekhnologiya.

[19] Sorokin B., Kvashnin G., Asafiev N., Kravchuk K., Luparev N., Sotnikov A. Microwave diamond-based HBAR as ultrathin film sensor. Pt deposition. Proceedings of the 2020 Joint Conference of the IEEE International Frequency Control Symposium and International Symposium on Applications of Ferroelectrics (IFCS-ISAF).

[20] Kvashnin G., Sorokin B., Asafiev N., Prokhorov V., Sotnikov A. (2022). Peculiarities of the acoustic wave propagation in diamond-based multilayer piezoelectric structures as “Me1/(Al, Sc)N/Me2/(100) diamond/Me3” and “Me1/AlN/Me2/(100) diamond/Me3” under metal thin film deposition. Electronics.

[21] Yamakawa Y., Sano K., Karasawa R., Yanagitani T. Broadband frequency viscositymeasurement using low TCF shear mode resonators consisting of c-axis tilted ScAlN thin film on thick AT-cut quartz plate. Proceedings of the 2017 19th International Conference on Solid-State Sensors, Actuators and Microsystems (TRANSDUCERS).

[22] Yanez J., Ledesma E., Uranga A., Barniol N. AlN-based HBAR ultrasonic sensor for fluid detection in microchannels with multi-frequency operation capability over the GHz range. Proceedings of the 2021 IEEE International Ultrasonics Symposium (IUS).

[23] Yanez J., Uranga A., Barniol N. Multi-Frequency Thin Film HBAR Microsensor for Acoustic Impedance Sensing Over the GHz Range. Proceedings of the 2021 21st International Conference on Solid-State Sensors, Actuators and Microsystems (Transducers).

[24] Barekatain M., Liu H., Kim E.S. (2022). Wireless and Battery-Less Tamper Detection with Pyroelectric Energy Converter and High-Overtone Bulk Acoustic Resonator. IEEE Sens. J..

[25] Patil A., Saha K. Stress Generation in Terfenol-D Using HBAR for NV Center Based Hybrid Sensor. Proceedings of the 2018 IEEE International Frequency Control Symposium (IFCS).

[26] Akiyama M., Kamohara T., Kano K., Teshigahara A., Takeuchi Y., Kawahara N. (2009). Enhancement of piezoelectric response in scandium aluminum nitride alloy thin films prepared by dual reactive cosputtering. Adv. Mater..

[27] Shvyd’ko Y., Stoupin S., Blank V., Terentyev S. (2011). Near-100% Bragg reflectivity of X-rays. Nat. Photonics.

[28] White R.M. (1987). A sensor classification scheme. IEEE Trans. Ultrason. Ferroel. Freq. Contr..

[29] Vig J.R., Walls F.L. A review of sensor sensitivity and stability. Proceedings of the 2000 IEEE/EIA International Frequency Control Symposium and Exhibition (Cat. No.00CH37052).

[30] Campanella H., Esteve J., Montserrat J., Uranga A., Abadal G., Barniol N., Romano-Rodríguez A. (2006). Localized and distributed mass detectors with high sensitivity based on thin-film bulk acoustic resonators. Appl. Phys. Lett..

[31] Sorokin B.P., Kvashnin G.M., Telichko A.V., Gordeev G.I., Burkov S.I., Blank V.D. (2015). Study of High-overtone Bulk Acoustic Resonators based on the Me1/AlN/Me2/(100) diamond piezoelectric layered structure. Acoust. Phys..

[32] Truell R., Elbaum C., Chick B.B. (1969). Ultrasonic Methods in Solid State Physics.

